# Targeting innate immunity to overcome immune evasion in HPV-associated cancers

**DOI:** 10.3389/fcimb.2026.1875111

**Published:** 2026-07-07

**Authors:** Xianguang Bai, Hongmei Dai, Zebao Lu, Shengnan Yu

**Affiliations:** 1 School of Medical Science and Technology, Pingdingshan University, Pingdingshan, China; 2 School of Medicine, Pingdingshan University, Pingdingshan, China; 3 School of Medical Technology, Chuxiong Medical College, Chuxiong, China; 4 Department of Oncology, Laboratory of Immunity, Inflammation & Cancer, The First Affiliated Hospital of Chongqing Medical University, Chongqing, China

**Keywords:** cGAS-STING, HPV-associated cancers, immune checkpoint evasion, innate immunity, interferon, PD-1, PD-L1, tumor microenvironment

## Abstract

Human papillomavirus (HPV)-associated cancers provide a unique model for understanding the paradox of viral antigenicity and tumor immune escape. Although viral oncoproteins such as E6 and E7 generate non-self antigens, many HPV-associated tumors persist under immune pressure and show heterogeneous responses to immune checkpoint blockade. This discrepancy reflects a process in which persistent HPV infection and malignant transformation remodel innate immune sensing, interferon (IFN) signaling, antigen presentation, and the tumor microenvironment. These changes impair dendritic cell activation and cytotoxic immune priming while promoting chronic inflammation, myeloid polarization, T-cell exhaustion, and PD-1/PD-L1-mediated adaptive immune resistance. In this review, we discuss how HPV-associated cancers subvert antiviral innate immunity and how these processes contribute to immune evasion. We further highlight therapeutic strategies aimed at restoring antiviral antitumor immunity, including immune checkpoint blockade, STING agonists, therapeutic HPV vaccines, radiotherapy-based combinations, TGF-β pathway inhibition, and biomarker-guided treatment approaches. Understanding the links among viral pathogenesis, innate immune remodeling, and checkpoint evasion may support more rational immunotherapy combinations for HPV-associated malignancies.

## 1 Introduction

Virus-associated cancers occupy a distinct position in tumor immunology. Unlike tumors driven mainly by endogenous mutations, these malignancies arise in the setting of persistent infection and often retain viral antigens that are foreign to the host (MacLennan and Marra, 2023; Jensen et al., 2024). Human papillomavirus (HPV)-associated cancers are among the most representative examples (Oyouni, 2023). High-risk HPV infection is etiologically linked to cervical cancer and contributes to a substantial fraction of anal, vulvar, vaginal, penile, and oropharyngeal cancers (Williamson, 2023). In these tumors, viral oncoproteins such as E6 and E7 promote malignant transformation by disrupting p53 and retinoblastoma protein pathways, inducing genomic instability, and sustaining proliferative signaling (Shin et al., 2012; Al Moustafa, 2015; Ren et al., 2020; Skelin et al., 2022). At the same time, the continued expression of viral antigens creates an apparent immunological vulnerability (Shamseddine et al., 2021).

This vulnerability generates a central paradox. HPV-associated tumors are virally antigenic and should theoretically be susceptible to immune recognition, yet they frequently evade immune surveillance. Persistent HPV infection can remain clinically silent for years before malignant progression (Song et al., 2015). Even after cancer develops, immune checkpoint inhibitors benefit only a subset of patients. In cervical cancer, PD-1/PD-L1 blockade has become an important therapeutic strategy in recurrent, metastatic, and locally advanced settings, but primary resistance and incomplete responses remain common (Liu et al., 2019; Huang et al., 2022; Tewari et al., 2022; Li et al., 2023). Similar patterns are observed in other HPV-associated malignancies, where viral antigen expression does not guarantee durable antitumor immunity (Xu et al., 2020; Zhang et al., 2022; Okuyama et al., 2025).

One explanation is that HPV-associated cancers are not merely antigen-positive tumors; they are immune-edited tumors shaped by long-term interaction between viral persistence, host sensing pathways, and the tumor microenvironment (TME) (Wang et al., 2019). Innate immune pathways are central to this process. Pattern-recognition receptors detect viral nucleic acids and cellular stress signals, activating interferon (IFN) responses, inflammatory cytokines, dendritic cell (DC) maturation, and adaptive immune priming (Li and Wu, 2021; Carroll et al., 2024). However, HPV has evolved mechanisms to dampen or redirect these antiviral programs (Lo Cigno et al., 2020). During tumor evolution, these alterations can be reinforced by oncogenic signaling, stromal remodeling, metabolic stress, and chronic inflammation (Binnewies et al., 2018; Shamseddine et al., 2021; Yuan et al., 2021; Hanahan, 2022). The result is a TME that may contain viral antigens and inflammatory signals but still suppress effective cytotoxic immunity.

Checkpoint evasion therefore needs to be understood within this broader context. PD-L1 expression can be induced by IFN-γ as part of adaptive immune resistance, by oncogenic signaling pathways, or by innate inflammatory circuits (Cha et al., 2019). T-cell exhaustion may result from chronic antigen exposure, inadequate DC priming, suppressive myeloid cells, and TGF-β-mediated immune exclusion (Yi et al., 2022b; Wang et al., 2025; Yin et al., 2025). These processes are closely linked to altered innate sensing and IFN remodeling (Kasuga et al., 2021; Zhang et al., 2023; Wu et al., 2024). Thus, targeting checkpoint molecules alone may be insufficient if the upstream immune architecture remains dysfunctional.

**Figure 1:**
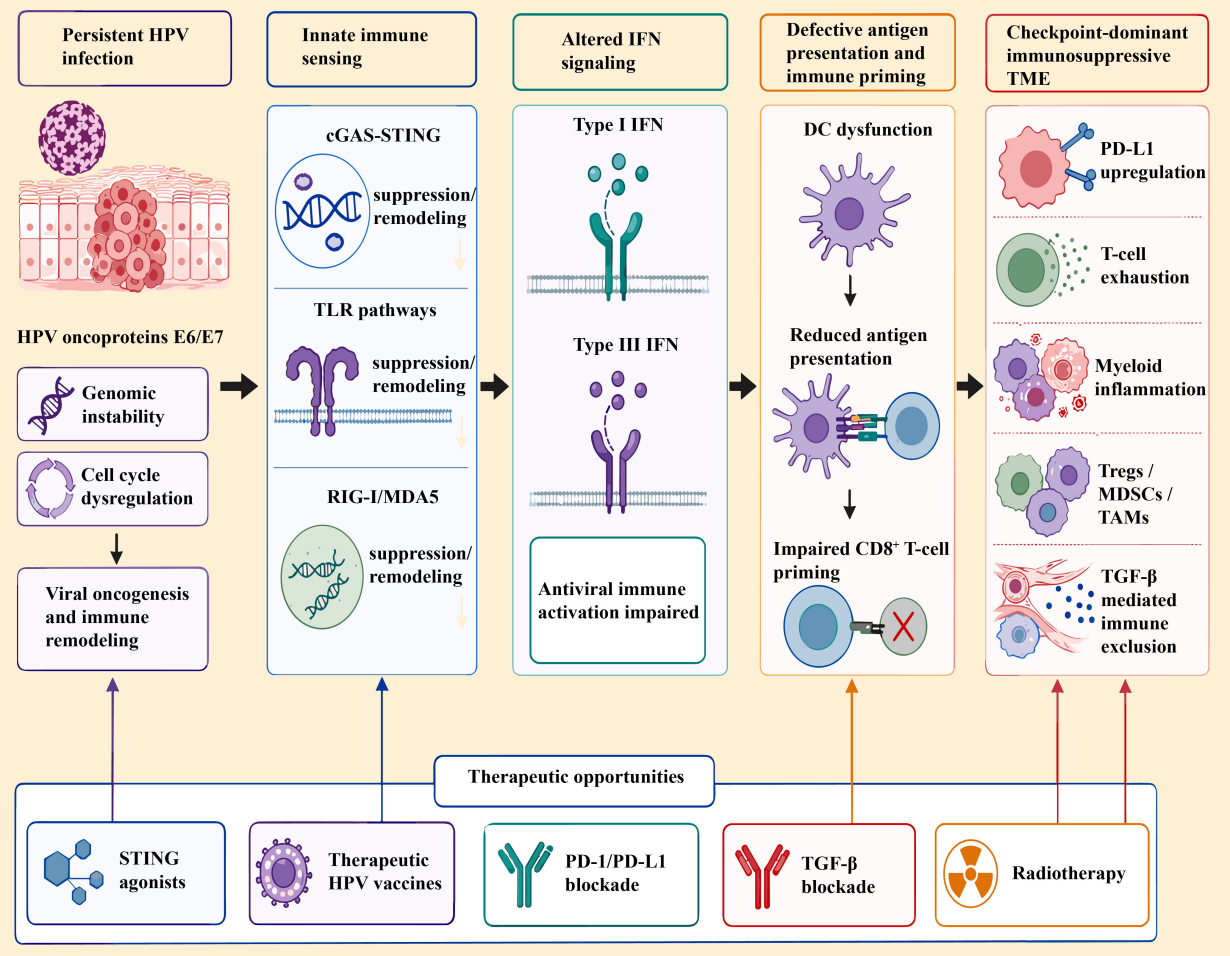
Innate immune remodeling drives checkpoint evasion in HPV-associated cancers. Persistent high-risk HPV infection promotes the continued expression of viral oncoproteins E6 and E7, which contribute to genomic instability, cell-cycle dysregulation, and progressive immune remodeling. During this process, innate immune sensing pathways, including cGAS-STING, Toll-like receptor (TLR) pathways, and RIG-I/MDA5-mediated RNA sensing, become suppressed or rewired, resulting in altered type I and type III interferon signaling. These changes impair antiviral immune activation, disrupt dendritic cell (DC) function, reduce antigen presentation, and weaken CD8^+^ T-cell priming. The downstream consequence is the establishment of a checkpoint-dominant immunosuppressive tumor microenvironment (TME), characterized by PD-L1 upregulation, T-cell exhaustion, myeloid inflammation, accumulation of immunosuppressive cells such as regulatory T cells (Tregs), myeloid-derived suppressor cells (MDSCs), and tumor-associated macrophages (TAMs), as well as TGF-β-mediated immune exclusion. Together, these events drive checkpoint evasion and immunotherapy resistance in HPV-associated cancers. Potential therapeutic opportunities include STING agonists, therapeutic HPV vaccines, PD-1/PD-L1 blockade, TGF-β blockade, and radiotherapy- or chemoradiotherapy-based strategies. This image distinguishes productive antiviral innate immune activation, which supports antigen presentation and T-cell priming, from checkpoint-dominant immune suppression, which promotes T-cell exhaustion, myeloid suppression, and immune escape.

This review focuses on the mechanistic connections between HPV-driven pathogenesis, innate immune remodeling, IFN signaling, and immune checkpoint evasion. We first summarize how HPV persistence and malignant transformation reshape host immune recognition. We then discuss cGAS-STING and other innate sensing pathways, the dual role of IFN signaling, and the downstream formation of checkpoint-dominant immune suppression. Finally, we consider therapeutic strategies that aim to restore antiviral antitumor immunity by combining checkpoint blockade with innate immune activation, therapeutic vaccination, TME remodeling, and biomarker-guided patient selection (**Figure 1**).

Although HPV-associated malignancies share a common viral etiology, important differences exist among cervical, anal, vulvar, vaginal, penile, and HPV-positive head and neck cancers. These differences involve anatomical location, epithelial barrier biology, microbiome exposure, immune-cell composition, treatment modality, and responsiveness to immunotherapy. For example, HPV-positive head and neck cancers often display a relatively T-cell-inflamed phenotype, whereas cervical cancers may be more strongly influenced by local mucosal immunity, stromal remodeling, and TGF-β-associated immune exclusion. Therefore, this review focuses primarily on shared immune-evasion principles across HPV-associated malignancies while acknowledging disease-specific heterogeneity where relevant.

## 2 HPV persistence, viral oncogenesis, and immune editing

HPV-associated carcinogenesis is initiated by infection of basal epithelial cells. Most infections are cleared by host immunity, but persistent infection with high-risk HPV types can lead to premalignant lesions and invasive cancer (Doorbar et al., 2012). Viral persistence is therefore not a passive event (Bodily and Laimins, 2011). It reflects successful evasion of innate and adaptive immune responses at the epithelial barrier. HPV maintains a relatively non-lytic life cycle, limits overt inflammation, and coordinates viral gene expression with epithelial differentiation (Zhou et al., 2019). This strategy reduces acute danger signals and allows infected cells to persist long enough for oncogenic events to accumulate.

The E6 and E7 oncoproteins are central to malignant transformation (Malla and Kamal, 2021). E6 promotes degradation of p53 and interferes with apoptosis, DNA damage responses, and innate antiviral signaling. E7 inactivates the retinoblastoma pathway, drives cell-cycle progression, and contributes to genomic instability (Hoppe-Seyler et al., 2018; Peng et al., 2024). These canonical oncogenic functions are accompanied by immunological effects. HPV oncoproteins can alter IFN-regulated gene expression, reduce antigen presentation, affect chemokine production, and impair the maturation or recruitment of antigen-presenting cells (Venuti et al., 2019; James et al., 2020; Gusho and Laimins, 2021; Challagundla et al., 2023; Bonfill-Teixidor et al., 2024). As a result, viral oncogenesis and immune escape are intertwined from the earliest stages of disease.

The presence of viral antigens also imposes immune selection (Krishna et al., 2018). E6- and E7-expressing cells may be recognized by HPV-specific T cells, but lesions that progress are likely to have acquired mechanisms that weaken immune detection or resist immune elimination (Keskin et al., 2011; Ramos et al., 2013). These mechanisms include loss or downregulation of antigen-processing machinery, impaired major histocompatibility complex expression, defective DC activation, enrichment of regulatory T cells, accumulation of suppressive myeloid populations, and upregulation of inhibitory ligands (Matthews et al., 2003; Mehta et al., 2008; Liu et al., 2017). Over time, HPV-associated cancers may become products of immune editing: they retain viral oncogene expression because it is required for malignant maintenance, but they remodel the immune environment to tolerate or conceal this vulnerability (Rampias et al., 2009; Magaldi et al., 2012).

This concept helps explain why HPV status alone is an incomplete biomarker (Tosi et al., 2022). HPV-positive tumors often show higher immune infiltration than virus-negative tumors in some anatomical sites, but immune infiltration may coexist with exhaustion, spatial exclusion, or suppressive cytokine programs (Partlová et al., 2015; Gameiro et al., 2018; Hladíková et al., 2018; Mariathasan et al., 2018). The clinical meaning of viral antigenicity depends on whether antigen presentation, innate activation, T-cell priming, and effector trafficking remain intact (Keskin et al., 2011; Lu et al., 2018). Therefore, a mechanistic framework for HPV-associated cancers must connect viral persistence to host immune remodeling rather than treating viral antigen expression as equivalent to immunogenicity.

## 3 Innate immune sensing pathways in HPV-associated tumors

Innate immune sensing provides the first line of defense against viral infection and shapes subsequent adaptive immunity (Iwasaki and Medzhitov, 2015). In HPV-associated cancers, the most relevant pathways include cytosolic DNA sensing through cGAS-STING, Toll-like receptors, RNA-sensing receptors such as RIG-I-like receptors, and inflammasome-related circuits (Zhou et al., 2013; Rattay et al., 2022; Shen et al., 2023). These pathways converge on type I and type III IFN production, NF-κB-driven inflammation, DC activation, and chemokine gradients that determine immune cell recruitment.

### 3.1 cGAS-STING signaling

The cGAS-STING pathway detects cytosolic DNA and activates TBK1, IRF3, NF-κB, and downstream IFN and inflammatory programs (Kwon and Bakhoum, 2020; Yi et al., 2021a; Wang et al., 2025). In principle, HPV infection and tumor-associated genomic instability could provide stimuli for DNA sensing (Lau et al., 2015; Mackenzie et al., 2017). However, many HPV-associated tumors exhibit impaired or context-dependent STING pathway activity (Lou et al., 2023). HPV oncoproteins have been reported to interfere with components of DNA sensing and IFN induction, thereby reducing antiviral immune activation (Bortnik et al., 2021). Tumor cells may also silence pathway components through epigenetic mechanisms or select for reduced innate immune visibility during progression (Falahat et al., 2021; Low et al., 2022).

Loss of effective cGAS-STING signaling can weaken DC activation, cross-presentation, and T-cell priming (Woo et al., 2014). This is particularly important because tumor-intrinsic sensing and host myeloid sensing cooperate to generate antitumor immunity (Deng et al., 2014). When tumor cells fail to produce adequate IFN or inflammatory cues, dying tumor cells may be less efficiently converted into immunogenic material for DCs (Fuertes et al., 2011). Importantly, the biological consequences of STING activation are highly context dependent. Acute and transient STING activation generally promotes type I IFN production, DC maturation, antigen cross-presentation, and cytotoxic T-cell priming. In contrast, chronic or dysregulated STING signaling may sustain inflammatory circuits, promote suppressive myeloid recruitment, induce counter-regulatory checkpoint pathways, or contribute to metastatic progression in certain settings (Bakhoum et al., 2018; Li et al., 2023). Therefore, the therapeutic goal is not simply to maximize STING signaling, but to restore productive innate activation that supports antigen presentation and cytotoxic immunity.

### 3.2 Toll-like receptors and RNA-sensing pathways

Although HPV is a DNA virus, endosomal and cytosolic pattern-recognition receptor networks beyond cGAS-STING also contribute to antiviral surveillance. TLR9 can recognize viral DNA, while TLR3, TLR7, TLR8, RIG-I, and MDA5 participate in broader antiviral immune responses and inflammatory crosstalk (Alexopoulou et al., 2001; Heil et al., 2004; Yoneyama et al., 2004; Hasan et al., 2007; Karim et al., 2013). In epithelial tissues, these pathways influence the production of cytokines, and chemokines that recruit and activate immune cells (Ferreira et al., 2020). HPV can dampen these signals to maintain persistence and limit immune-mediated clearance (Hasan et al., 2013).

The relevance of these pathways extends to cancer therapy. TLR agonists and RNA-sensing agonists can activate DCs, enhance antigen presentation, and promote T-cell infiltration (Lövgren et al., 2017; Takeda et al., 2017). However, their effects depend on dose, route, tumor site, and the pre-existing TME (Everson et al., 2024). In HPV-associated cancers, where viral antigens are present but immune activation is often insufficient, PRR agonists may help convert antigenicity into effective immunogenicity (Daayana et al., 2010; Ferris et al., 2018; Koerner et al., 2021). Still, activation of innate pathways can also induce counter-regulatory checkpoint molecules, including PD-L1, creating a rationale for combining innate immune agonists with checkpoint blockade (Fernandez-Rodriguez et al., 2023).

### 3.3 DCs and antigen presentation

DCs are the critical bridge between innate sensing and adaptive immunity. Productive recognition of viral or tumor-derived nucleic acids induces DC maturation, type I IFN production, antigen cross-presentation, and priming of CD8^+^ T cells (Lv et al., 2020). In HPV-associated tumors, defects at this level can uncouple viral antigen expression from effective T-cell responses (Mehta et al., 2008). Reduced DC abundance, impaired maturation, tolerogenic programming, or exclusion from tumor nests can all limit antitumor immunity (Mayoux et al., 2020).

Antigen presentation defects may occur in tumor cells and antigen-presenting cells (Leone et al., 2013). Tumor cells can downregulate human leukocyte antigen molecules, antigen-processing machinery, or IFN responsiveness (Hasim et al., 2012; Gao et al., 2016). DCs can become suppressed by lactate, TGF-β, IL-10, prostaglandins, or myeloid-derived suppressive networks (Ghiringhelli et al., 2005; Gottfried et al., 2006; Ahmadi et al., 2008). As a result, T cells may be exposed to chronic antigen without adequate costimulation or may fail to enter tumor regions where malignant cells reside. These defects create a permissive environment for checkpoint evasion because PD-1/PD-L1 blockade depends on the presence of pre-existing or restorable T-cell immunity.

## 4 IFN signaling: antiviral defense and adaptive immune resistance

IFN signaling has a dual role in HPV-associated cancers. Acute IFN responses are essential for antiviral defense and antitumor immunity (Diamond et al., 2011; Benci et al., 2019; Castro-Muñoz et al., 2022). Type I and type III IFN can inhibit viral replication, enhance antigen processing and presentation, activate DCs, support NK-cell function, and facilitate T-cell priming (Lazear et al., 2019; Mesev et al., 2019). In early infection, robust IFN responses favor viral clearance and reduce the probability of persistence.

HPV persistence is associated with attenuation of these antiviral programs (Ronco et al., 1998; Bachmann et al., 2002). Viral oncoproteins can interfere with IFN production and downstream signaling, allowing infected cells to escape immune detection (Zhou et al., 2013; Poirson et al., 2022). In established tumors, impaired IFN responsiveness can reduce antigen presentation and weaken recruitment of effector lymphocytes (Zhou et al., 2013; Benci et al., 2016). Tumors with low IFN activity may resemble immune-cold or immune-excluded phenotypes, even when viral antigens are present (Ayers et al., 2017).

At the same time, IFN signaling can also promote adaptive immune resistance. IFN-γ produced by activated T cells induces PD-L1 expression on tumor cells and myeloid cells (Taube et al., 2012; Lyford-Pike et al., 2013). IFN-regulated programs may also increase IDO1, chemokines, and other immunoregulatory molecules (Jürgens et al., 2009). This response initially reflects immune recognition, but it can become a mechanism of immune escape if inhibitory feedback dominates. Chronic or spatially restricted IFN signaling may therefore generate a TME that is inflamed but functionally suppressed (Chen et al., 2022).

This duality is particularly relevant for therapeutic design. Productive IFN signaling may enhance antigen presentation and immune priming, whereas chronic or spatially restricted IFN activity may reinforce checkpoint-mediated suppression. Therefore, the timing, cellular source, and intensity of IFN signaling should be considered when combining innate immune activation with checkpoint blockade.

## 5 Checkpoint evasion driven by innate immune remodeling

Immune checkpoint evasion in HPV-associated cancers arises from the convergence of chronic viral antigen exposure, altered innate sensing, IFN remodeling, antigen-presentation defects, and suppressive TME formation (Zhou et al., 2019). The PD-1/PD-L1 axis is the best-characterized pathway, but other inhibitory receptors and ligands, including CTLA-4, TIM-3, LAG-3, TIGIT, and VISTA, may also contribute to T-cell dysfunction (Hladíková et al., 2018; Wuerdemann et al., 2020; Zou et al., 2023).

### 5.1 PD-L1 induction and adaptive immune resistance

PD-L1 expression in HPV-associated tumors can arise through multiple mechanisms (Rotman et al., 2020). Inflammatory induction by IFN-γ reflects an adaptive response to T-cell attack (Lyford-Pike et al., 2013). Oncogenic pathways such as PI3K-AKT, EGFR signaling, hypoxia, and inflammatory NF-κB programs can also contribute (Lin et al., 2023). In virus-associated cancers, PD-L1 may therefore indicate either an inflamed TME with ongoing immune recognition or a tumor-intrinsic immune escape program (Mezache et al., 2015). This complexity explains why PD-L1 expression is clinically useful in some settings but remains an imperfect biomarker.

The link between innate immune activation and PD-L1 induction has therapeutic implications (Pan et al., 2020; Yi et al., 2022a; Li et al., 2024). STING or TLR agonists may increase tumor inflammation and improve T-cell recruitment, but they may also increase PD-L1 expression as part of an IFN-driven feedback loop (Sato-Kaneko et al., 2017; Gan et al., 2021; Kaur et al., 2022; Choi et al., 2024). Rather than viewing this as a negative effect, it can be interpreted as a rationale for rational combination therapy. Innate activation may provide the priming and infiltration signals, while PD-1/PD-L1 blockade prevents newly induced inhibitory feedback from limiting effector function (Zhang et al., 2023; Pulliam et al., 2024; Yi et al., 2026).

### 5.2 T-cell exhaustion and chronic antigen exposure

HPV-associated tumors present persistent viral antigens, and chronic antigen exposure can drive T-cell exhaustion (Utzschneider et al., 2016; Krishna et al., 2018). Exhausted T cells are not simply inactive; they exist along a spectrum that includes progenitor-like, transitional, and terminally exhausted states (Jadhav et al., 2019; Miller et al., 2019). Checkpoint blockade is most effective when a pool of reinvigoratable T cells remains (Pauken et al., 2016). If HPV-specific or tumor-specific T cells are terminally exhausted, spatially excluded, or insufficiently primed, PD-1 blockade alone may have limited efficacy (Garris et al., 2018).

Innate immune remodeling shapes this exhaustion trajectory. Inadequate DC activation can lead to poor priming and dysfunctional T-cell differentiation. Suppressive cytokines and myeloid cells can limit effector expansion. TGF-β can prevent T cells from entering tumor nests or sustaining cytotoxic activity (Thomas and Massagué, 2005; Mariathasan et al., 2018). Therefore, reversing T-cell exhaustion may require more than checkpoint release. It may require restoration of antigen presentation, inflammatory priming, and tissue access.

### 5.3 Myeloid inflammation and immune exclusion

Virus-associated inflammation does not necessarily translate into productive antitumor immunity. Chronic inflammation can recruit monocytes, macrophages, neutrophils, and myeloid-derived suppressor cells that produce IL-10, TGF-β, arginase, reactive oxygen species, prostaglandins, and other suppressive mediators (Kusmartsev et al., 2004; Corzo et al., 2009; Srivastava et al., 2010; Li et al., 2021). These cells can inhibit T-cell activation, promote angiogenesis, remodel extracellular matrix, and create immune-excluded niches.

Myeloid inflammation is closely related to innate sensing (Liu et al., 2025). Depending on context, PRR and IFN pathways can promote either immunostimulatory DC programs or suppressive myeloid circuits. In HPV-associated cancers, the balance between these states may determine whether viral antigenicity becomes clinically meaningful (Kansy et al., 2023). Therapeutic strategies that activate innate immunity should therefore aim to favor DC-mediated T-cell priming and avoid reinforcing suppressive myeloid inflammation (Lam et al., 2021; Nguyen et al., 2024).

TGF-β is another important mediator of immune exclusion. It can restrict cytotoxic lymphocyte infiltration, promote stromal activation, support regulatory T cells, and dampen effector functions (Li et al., 2020; Reid et al., 2024; George et al., 2025). In tumors with strong TGF-β signatures, checkpoint blockade may fail because T cells cannot access tumor nests or remain functionally suppressed (Mariathasan et al., 2018; Tauriello et al., 2018; Chu et al., 2023). Combining checkpoint blockade with TGF-β pathway inhibition is therefore conceptually attractive, particularly in immune-excluded HPV-associated tumors.

## 6 Therapeutic strategies targeting innate immune remodeling

The goal of targeting innate immune remodeling is to convert HPV-associated tumors from antigen-positive but immune-suppressed lesions into tumors capable of productive antiviral antitumor immunity. Several therapeutic strategies can be organized around this goal (**Table 1**).

**Table 1:** Innate immune remodeling mechanisms that contribute to checkpoint evasion in HPV-associated cancers.

Mechanisms	HPV-related driver or host alteration	Immune consequence	Therapeutic implication
Suppressed DNA sensing	High-risk HPV oncoproteins and tumor evolution can attenuate cGAS-STING signaling and downstream IRF3/NF-kappaB activation.	Reduced type I IFN production, weaker dendritic-cell activation, and impaired cross-priming of CD8^+^ T cells.	STING agonists or radiotherapy-based innate activation may restore immune priming when pathway competence is retained.
Altered PRR networks	HPV persistence may dampen TLR- and RNA-sensing programs at epithelial barriers and in antigen-presenting cells.	Insufficient inflammatory cues and chemokines for immune recruitment, especially in early or immune-cold lesions.	TLR or RIG-I-like receptor agonists may be combined with ICB or HPV vaccines to convert antigenicity into immunogenicity.
Interferon remodeling	Acute antiviral IFN signaling is weakened, whereas chronic or spatially restricted IFN programs may persist in established tumors.	Coexistence of defective antigen presentation and inflammatory feedback signals.	Therapy should restore productive IFN signaling while blocking compensatory PD-1/PD-L1 feedback.
Antigen-presentation defects	Tumor immune editing may select for reduced HLA expression, impaired antigen-processing machinery, or low IFN responsiveness.	Viral E6/E7 antigens are present but incompletely displayed to cytotoxic T cells.	Biomarker-guided use of IFN-restoring agents, epigenetic therapy, vaccines, or adoptive T-cell approaches may be considered.
Dendritic-cell dysfunction	Suppressive TME signals such as lactate, IL-10, TGF-β, prostaglandins, and myeloid inflammation restrain DC maturation.	Defective cross-presentation and weak expansion of HPV-specific CD8^+^ T cells.	STING agonists, TLR agonists, CD40 agonists, radiotherapy, or vaccine adjuvants may enhance DC-mediated priming.
Myeloid inflammation	Persistent viral inflammation and tumor-derived cytokines recruit TAMs, neutrophils, and MDSCs with suppressive functions.	Arginase, ROS, IL-10, TGF-β, and prostaglandins inhibit T-cell activation and support immune exclusion.	Myeloid-targeted agents, anti-angiogenic therapy, or cytokine/chemokine blockade may improve ICB responsiveness.
TGF-β-driven immune exclusion	HPV-associated tumors may acquire stromal activation and TGF-beta-rich niches during progression.	Cytotoxic lymphocytes remain at the invasive margin or stromal compartment rather than entering tumor nests.	TGF-β blockade, bifunctional checkpoint/TGF-β targeting, or stromal remodeling may complement ICB.
Chronic viral antigen exposure	Persistent expression of E6/E7 maintains antigenic pressure over years of infection and tumor evolution.	HPV-specific T cells progress toward dysfunctional or exhausted states.	ICB should be paired with strategies that increase antigen presentation, T-cell priming, or progenitor-like T-cell pools.

DC, dendritic cell; HPV, human papillomavirus; ICB, immune checkpoint blockade; IFN, interferon; MDSC, myeloid-derived suppressor cell; PRR, pattern-recognition receptor; ROS, reactive oxygen species; TAM, tumor-associated macrophage; TGF-β, transforming growth factor-β.

### 6.1 Immune checkpoint blockade

PD-1/PD-L1 blockade is already incorporated into the clinical management of selected HPV-associated cancers, particularly cervical cancer, and is being explored across other HPV-associated malignancies. Its activity supports the concept that virally antigenic tumors can be susceptible to immune reinvigoration (Morris et al., 2017; Naumann et al., 2019; Lorusso et al., 2024). However, response heterogeneity indicates that checkpoint blockade requires a permissive immune context. Patients with pre-existing T-cell inflammation, preserved antigen presentation, and lower suppressive myeloid or stromal barriers may be more likely to benefit (Kalbasi and Ribas, 2020). Conversely, tumors with defective innate sensing, poor DC activation, TGF-β-driven exclusion, or terminal T-cell exhaustion may require combination strategies (Mellman et al., 2023).

CTLA-4 blockade and dual checkpoint inhibition may broaden T-cell priming and reduce regulatory T-cell suppression, but toxicity remains a concern (Gide et al., 2019; Paz-Ares et al., 2021). Bispecific antibodies that target two checkpoint pathways or combine checkpoint blockade with other immune-modulating functions may offer new opportunities (Feng et al., 2022; Lorusso et al., 2024). In HPV-associated tumors, the ideal checkpoint strategy should be selected according to the dominant immune defect: insufficient priming, T-cell exhaustion, immune exclusion, or suppressive myeloid inflammation.

### 6.2 STING agonists and innate immune activation

Compared with immune checkpoint blockade, STING agonists remain largely at the preclinical or early clinical development stage. STING agonists are particularly relevant for HPV-associated cancers because they directly target a pathway linked to DNA sensing, IFN induction, DC activation, and T-cell priming. Preclinical studies suggest that STING activation can inflame immune-cold tumors, increase chemokine production, promote DC maturation, and enhance CD8^+^ T-cell infiltration (Fu et al., 2015; Jing et al., 2019). In HPV-associated tumors with suppressed DNA sensing, STING agonists may help restore antiviral immune recognition (Lou et al., 2023).

Nevertheless, several challenges remain. Systemic STING activation can cause toxicity, and intratumoral delivery may not be feasible for all lesions. The timing, dose, and formulation of STING agonists may determine whether they generate productive immunity or excessive inflammatory feedback (Wu et al., 2020; Wang et al., 2024; Yang et al., 2024). STING activation may also induce PD-L1 expression, supporting combination with PD-1/PD-L1 blockade. Biomarkers of STING pathway competence, DC abundance, IFN responsiveness, and baseline immune infiltration may be needed to identify patients most likely to benefit.

### 6.3 Therapeutic HPV vaccines and antigen-directed immunity

Therapeutic vaccines targeting HPV E6 and E7 aim to amplify virus-specific T-cell responses. This strategy is biologically appealing because E6 and E7 are tumor-specific, continuously expressed, and required for malignant maintenance (Magaldi et al., 2012). Vaccine platforms include peptides, proteins, DNA, RNA, viral vectors, bacterial vectors, and DC-based approaches (Skeate et al., 2016; Lei et al., 2025). Therapeutic HPV vaccines have demonstrated immunogenicity in clinical studies, but their clinical efficacy remains variable and they have not yet achieved broad routine adoption for advanced HPV-associated cancers (Kenter et al., 2008).

One reason is that vaccine-induced T cells must enter and function within a suppressive TME. If antigen presentation is defective, DCs are suppressed, or PD-L1 and TGF-β pathways dominate, vaccine responses may not translate into tumor regression. Therefore, therapeutic vaccines may be most effective when combined with checkpoint blockade, innate immune agonists, radiotherapy, or TME-modulating agents (Massarelli et al., 2019). In premalignant lesions or minimal residual disease settings, where immune suppression may be less entrenched, therapeutic vaccination may have greater potential.

### 6.4 Radiotherapy, chemoradiotherapy, and innate immune priming

Radiotherapy can induce immunogenic cell death, release tumor antigens, generate cytosolic DNA, and activate cGAS-STING-dependent IFN signaling (Gameiro et al., 2014). These effects provide a strong rationale for combining radiotherapy with checkpoint blockade in HPV-associated cancers (Ngiow et al., 2015; Twyman-Saint Victor et al., 2015). Among combination strategies, chemoradiotherapy-based immunotherapy is among the most clinically advanced approaches in cervical cancer, although optimal sequencing and patient selection remain areas of active investigation (Lorusso et al., 2024).

Radiation-induced innate activation is context-dependent (Vanpouille-Box et al., 2017). High-dose or repeated radiation may also induce immunosuppressive pathways, recruit myeloid cells, or cause lymphodepletion. The therapeutic window may depend on fractionation, timing of checkpoint blockade, tumor burden, and baseline immune state (Sia et al., 2021; Bergerud et al., 2024). For HPV-associated tumors, radiotherapy may be particularly useful when it increases viral and tumor antigen release while simultaneously activating innate sensing pathways that support T-cell priming (Reits et al., 2006).

### 6.5 TGF-β blockade and remodeling of immune exclusion

TGF-β pathway inhibition remains an active area of translational and clinical investigation and offers a complementary approach to innate immune activation. While STING agonists, vaccines, and radiotherapy may increase immune priming and infiltration signals, TGF-β blockade may help overcome stromal barriers and immune exclusion (Yi et al., 2021b; Lan et al., 2022; Rajan et al., 2024; Yi et al., 2024). This strategy is relevant for tumors in which cytotoxic lymphocytes are present at the margin but fail to penetrate tumor nests. Combining TGF-β inhibition with PD-1/PD-L1 blockade may therefore be especially useful in immune-excluded HPV-associated cancers (Birrer et al., 2024).

However, TGF-β has broad physiological functions, and pathway inhibition can carry toxicity. Patient selection is essential (Deng et al., 2024). Spatial immune profiling, TGF-β gene signatures, fibroblast activation markers, and patterns of T-cell exclusion may help identify tumors most likely to benefit. Rational combinations should aim to restore immune access and effector function without causing excessive inflammation or tissue damage.

## 7 Biomarkers and future perspectives

**Figure 2:**
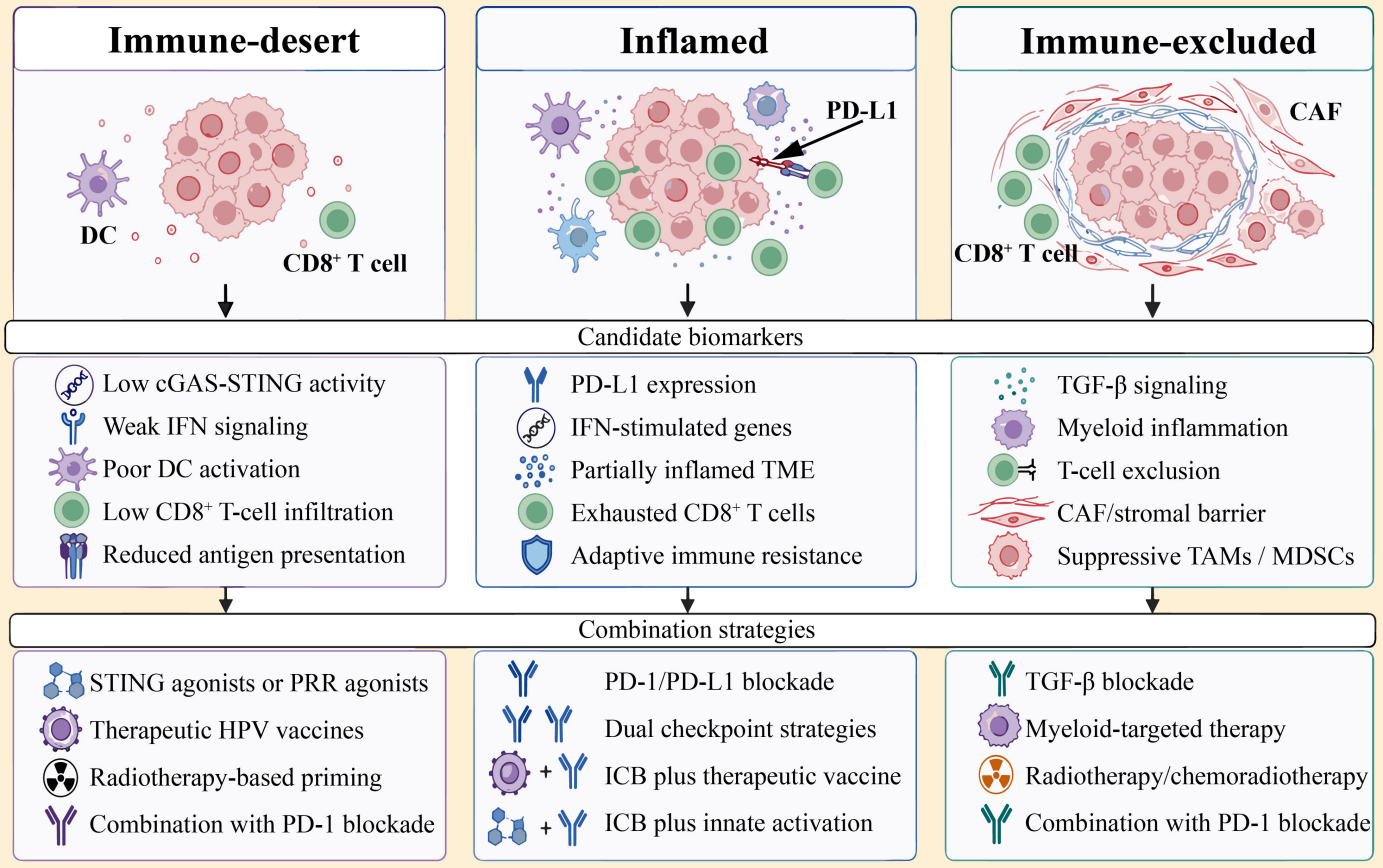
Biomarker-guided strategies to restore antiviral antitumor immunity in HPV-associated cancers. HPV-associated cancers may be broadly categorized into distinct immune states that can inform rational combination immunotherapy. The immune-desert/innate-low state is characterized by low cGAS-STING activity, weak type I/III interferon signaling, poor dendritic cell activation, low CD8^+^ T-cell infiltration, and reduced antigen presentation, suggesting potential benefit from STING agonists or other pattern-recognition receptor (PRR) agonists, therapeutic HPV vaccines, radiotherapy-based priming, and combination approaches with PD-1/PD-L1 blockade. The inflamed but suppressed state shows PD-L1 expression, interferon-stimulated gene signatures, partially inflamed TME features, exhausted CD8^+^ T cells, and adaptive immune resistance, supporting the use of PD-1/PD-L1 blockade, dual checkpoint strategies, or combinations with therapeutic vaccination or innate immune activation. The immune-excluded/stromal-restrained state is marked by TGF-β signaling, myeloid inflammation, T-cell exclusion, cancer-associated fibroblast (CAF)-mediated stromal barriers, and suppressive TAM/MDSC infiltration, indicating potential benefit from TGF-β blockade, myeloid-targeted approaches, radiotherapy/chemoradiotherapy, and combination regimens with PD-1/PD-L1 blockade.

Current biomarker approaches in HPV-associated cancers remain incomplete. PD-L1 expression and HPV status provide useful information but cannot fully capture the immune state of the tumor. A more integrated framework is needed. Such a framework should include viral antigen expression, cGAS-STING pathway activity, IFN gene signatures, antigen-presentation capacity, DC states, T-cell exhaustion profiles, myeloid inflammation, TGF-β-driven exclusion, and spatial organization of immune cells (**Figure 2**) (Hegde et al., 2016).

From a translational perspective, HPV-associated tumors may be broadly categorized into several representative immune states. Inflamed-responsive tumors contain pre-existing T-cell infiltration, preserved antigen presentation, and relatively limited suppressive barriers, and may be more likely to respond to checkpoint blockade (Chen and Mellman, 2017). Inflamed-suppressed tumors show immune infiltration but remain functionally constrained by T-cell exhaustion, PD-L1 induction, suppressive myeloid cells, or other adaptive resistance programs. Immune-excluded tumors are characterized by stromal barriers, TGF-β signaling, fibroblast activation, and restricted T-cell access to tumor nests. In contrast, innate-sensing-deficient tumors display impaired cGAS-STING or IFN signaling, poor DC activation, and weak effector-cell recruitment. These immune states are not mutually exclusive, but they provide a practical framework for linking biomarkers to therapeutic selection.

Circulating tumor HPV DNA may provide a dynamic marker of tumor burden and treatment response, particularly in cervical and HPV-positive head and neck cancers (Haring et al., 2021; Han et al., 2024). However, it should be integrated with tissue-based immune profiling rather than used in isolation. Single-cell sequencing, spatial transcriptomics, multiplex immunofluorescence, and functional assays can help distinguish inflamed-responsive tumors from inflamed-suppressed or immune-excluded tumors (Lee et al., 2024; Markovits et al., 2025). This distinction is critical because different immune states require different therapeutic interventions.

Future clinical trials should move beyond empiric combinations. Instead of combining checkpoint inhibitors with innate agonists, vaccines, radiotherapy, or TGF-β inhibitors in unselected populations, trials should be designed around mechanistic hypotheses and biomarker-defined immune states. For example, tumors with low innate sensing and poor T-cell infiltration may be prioritized for STING or TLR agonist combinations (Nishinakamura et al., 2025; Song et al., 2025). Tumors with viral antigen expression but exhausted T cells may benefit from checkpoint blockade plus therapeutic vaccination (Youn et al., 2020). Tumors with T-cell exclusion and stromal activation may require TGF-β or myeloid-targeted strategies. Such precision immunotherapy will require prospective validation but offers a path toward improving outcomes in HPV-associated cancers.

Another important direction is prevention and early intervention. Prophylactic HPV vaccination remains the most effective strategy for reducing HPV-associated cancer incidence (Lei et al., 2020). However, individuals with established persistent infection, premalignant lesions, or cancer require therapeutic approaches. Understanding how innate immune remodeling occurs during progression from infection to premalignancy and invasive disease may identify windows for intervention before immune escape becomes fixed.

## 8 Conclusion

HPV-associated cancers illustrate the paradox of viral immunogenicity and immune escape. Although these tumors express viral antigens that can be recognized by the immune system, persistent infection and malignant progression are accompanied by extensive remodeling of innate immune sensing, IFN signaling, antigen presentation, and the TME. These alterations promote checkpoint evasion, T-cell exhaustion, myeloid inflammation, and immune exclusion. Therefore, effective immunotherapy for HPV-associated cancers will likely require more than checkpoint blockade alone. Strategies that restore productive innate immune activation, enhance viral antigen-directed T-cell responses, and remodel suppressive microenvironments may help convert viral antigenicity into durable antitumor immunity. A deeper understanding of the viral-host-immune axis will support biomarker-guided combinations and more effective therapeutic strategies for HPV-associated malignancies.
